# Comparison between AJCC 8th prognostic stage and UICC anatomical stage in patients with primary breast cancer: a single institutional retrospective study

**DOI:** 10.1007/s12282-020-01115-x

**Published:** 2020-06-03

**Authors:** Ryo Tanaka, Yoji Yamagishi, Tomomi Koiwai, Takako Kono, Makiko Fukumura-Koga, Takahiro Einama, Tamio Yamasaki, Kimiya Sato, Hideki Ueno, Yoji Kishi, Hitoshi Tsuda

**Affiliations:** 1 Department of Basic Pathology, National Defense Medical College, 3-2 Namiki, Tokorozawa, Saitama 359-8513 Japan; 2 National Defense Medical College, 3-2 Namiki, Tokorozawa, Saitama 359-8513 Japan; 3 Department of Surgery, National Defense Medical College, 3-2 Namiki, Tokorozawa, Saitama 359-8513 Japan

**Keywords:** AJCC 8th edition, Prognostic stage, Primary breast cancer

## Abstract

**Background:**

The 8th edition American Joint Committee on Cancer (AJCC) proposed a prognostic stage (PS), which included not only anatomical factors, but also biological factors. We aimed to investigate the clinicopathological significance of the PS and to compare PS and anatomical stage (AS) that has been established by the Union for International Cancer Control (UICC).

**Methods:**

Between 2002 and 2017, 800 patients were included in the study. Patients were classified using pathological UICC AS and pathological AJCC PS. The usefulness of PS in comparison with AS was validated using the Akaike information criterion (AIC) and Harrell concordance index (C-index).

**Results:**

A total of 401 (50.1%) patients had pathological AS I, 324 (40.5%) had AS II, and 75 (9.4%) had AS III. Meanwhile, 535 (66.8%) had pathological PS I, 163 (20.4%) had PS II, and 102 (12.8%) had PS III. The number of AS II cases was 1.99-fold higher than that of PS II cases. For each stage, these survival curves were almost similar between AS and PS classification. Therefore, many patients to be classified into stage I and stage III were included in AS II group, while many patients to be classified into stage II were included in AS I group. To trichotomize the survival groups, PS appeared to be more specific than AS, and AIC and C-index confirmed the speculation.

**Conclusion:**

For the prognostication of primary breast cancer patients, AJCC PS appeared to be able to stratify the cases more appropriately than UICC AS.

**Electronic supplementary material:**

The online version of this article (10.1007/s12282-020-01115-x) contains supplementary material, which is available to authorized users.

## Introduction

Breast cancer is the most frequent malignant disease, the number of the patients who were newly diagnosed with breast cancer was 1.8 million, and 471,000 died of breast cancer in 2013 [1]. Tumor staging system codified by Union for International Cancer Control (UICC) started in 1933, and is maintained by both the UICC and American Joint Committee on Cancer (AJCC). This system was based on the anatomical factors [primary tumor (T), regional lymph node (N), and distant metastasis (M)], and called the TNM classification system or anatomical stage (AS).

The AS has been used worldwide for various purposes, e.g., standardization of treatment, and for comparison of patient’s outcomes based on the common criteria [2]. The primary objective of the UICC AS was to provide a name to an initially diagnosed breast cancer and prevent apparent useless therapy. The past recommendations for most systemic therapies, especially chemotherapy, had been based on the status of regional lymph nodes and the primary tumor size [3, 4].

However, in the era of worldwide use of endocrine therapy and molecular targeted therapy, the use of UICC AS alone does not appear sufficient to decide which kind of an adjuvant therapy or a neoadjuvant systemic therapy should be used. The evolving knowledge of breast cancer biology suggests that the status of tumor biomarkers should be documented by the time of initial therapy. These biomarkers included tumor grade, estrogen receptor (ER), progesterone receptor (PgR), and human epidermal growth factor receptor 2 (HER2), and are used for the prediction of prognosis. Tumor grade, which includes histological grade (HG) and nuclear grade (NG) [5–8], is the most reliable and widely used for evaluating tumor differentiation and for selecting the appropriate adjuvant chemotherapy. Hormone receptor status and HER2 status were also useful for predicting the therapeutic effect of the endocrine therapy and HER2 targeted therapy, respectively [9–12]. The selection of optimal treatment based on these biological factors has become more important [13].

American Joint Committee on Cancer (AJCC), 8th edition established a prognostic stage (PS) classification that not only included anatomical factors (T, N, and M), but also biological factors (tumor grade, ER, PgR, and HER2) in 2016 [2]. Using the PS classification, it may possible to choose more effective systemic therapies and to predict patient’s outcome more accurately compared with the use of T, N, and M factors only.

There are two widely used AS classifications: clinical AS is basically determined before the initial treatment. Pathological AS is determined based on the histopathological findings of surgically resected specimens. Likewise, in PS classification, two systems, clinical PS and pathological PS, are considered.

This study aimed to investigate the utility of pathological PS by comparing it with pathological AS in a single-institution cohort. Using the cohort, we statistically compared the fitness as a model and accuracy of prognosis predictability between pathological PS and pathological AS.

For tumor grading, it is recommended to use HG by the World Health Organization classification, 5th ed. [14], but in Japan, NG is also recommended in the General Rules for Clinical and Pathological Recording of Breast Cancer, 18th ed. [15]. Therefore, we classified breast cancer cases not only into PS using HG but also into PS using NG in order to reveal if there are differences in prognostic impact between pathological AJCC PS and pathological PS using NG.

## Materials and methods

### Eligible patients

Between January 2002 and December 2017, radical surgery was performed for the 1159 patients who were diagnosed as histologically primary carcinoma of the breast. Of these, 359 patients were excluded from this study because of (1) the history of malignant diseases other than breast cancer within 5 years (*n* = 32), (2) preoperative chemotherapy or endocrine therapy (*n* = 138), (3) previous treatment of ipsilateral or contralateral breast cancer (*n* = 108), (4) pStage 0 (*n* = 59) and Stage IV (*n* = 5), (5) pTis with node metastases (pN2a) (*n* = 1), (6) incomplete information of any one of pT, pN, M, ER, PgR, HER2, HG and NG (*n* = 115), and/or (7) HER2 equivocal cases (*n* = 11). Of these, 59 patients were excluded because of two or more condition of (1) through (7). Finally, 800 patients were eligible.

### Histological study

Two observers (H.T. or T.K. and Y.Y.) performed the pathological diagnosis. We prepared two PS classifications: one is PS incorporating HG (AJCC PS), and the other is PS incorporating NG instead of HG (PS-NG). HG was given according to Nottingham modification of the Scarff-Bloom-Richardson scoring system [16]. NG was determined by the sum of the nuclear atypia score and the mitosis count score [8]. From the studies of early breast cancers, the prognostic impact was similar between NG and HG [17]. ER and PgR were assessed by immunohistochemistry and defined as positive if 1% or higher of the constituent carcinoma cells were immunoreactive [18]. HER2 status was assessed according to the 2013 American Society of Clinical Oncology (ASCO)/College of American Pathologists (CAP) guidelines [19]. Ki-67 labeling index was judged as high if ≥ 14% and low if > 14% of cancer cells according to the Breast Cancer Working Group [20] [21]. Pathological stages, i.e., pathological AS and pathological PS, were determined based on the clinical and pathological recording of breast cancer recommended by the Union for International Cancer Control (UICC) 8th edition and AJCC 8th edition, respectively.

### Statistical analysis according to prognosis

Statistical differences were tested using the Chi-square test or Fisher’s exact test. The Kaplan–Meier curves for relapse-free survival (RFS) and overall survival (OS) were drawn, and their differences were tested using the log-rank test. The hazard ratio of the parameters of recurrence or death was calculated using the Cox’s univariate proportional hazard model. The independent significance of these parameters was tested using the Cox’s multivariate proportional hazard model. All statistical analyses were two sided, and a *P* value of < 0.05 was considered significant. For the comparison among three groups or more, the *P* values were adjusted using the Bonferroni correction.

To assess the goodness of fit of a model, the Akaike information criterion (AIC) was used [22]. The better model was considered to acquire a lower AIC value. The Harrell concordance index (C-index), which measures the proportion of pairs for which the predicted and observed outcomes are concordant, was used to measure the model’s prognosis predicting performance [23]. The model with a higher C-index was considered to have a better predictive performance. All statistical analyses were performed using JMP^®^ 14 (SAS Institute Inc., Cary, NC, USA).

### Ethical approval and consent to participate

This study was performed in accordance with the Declaration of Helsinki and was approved by the institutional review board of National Defense Medical College (registration number: 3003). All patients agreed to participate in this study, and a written informed consent was obtained from all patients.

## Results

### Patient characteristics

From the 800 patients, we acquired the data of sex, age, pathological tumor size, pathological tumor invasion size, pT, pN, lymphatic invasion, HG, NG, ER, PgR, HER2 status, Ki-67 labeling index, histological type, pathological UICC AS, pathological AJCC PS, pathological PS-NG, procedure (breast and axillary lymph node), medication therapy (endocrine therapy, chemotherapy, and anti-HER2 therapy), radiation therapy, relapse-free survival rate, and overall survival rate (Table 1). The pathological T factors were pT1 in 489 (61.1%), pT2 in 273 (34.1%), and pT3 in 38 (4.7%).

**Table 1:** Patients characteristics

Parameter	Number of cases (%)	
Total	800	(100.0)
Sex		
Male	6	(0.8)
Female	794	(99.2)
Age (year)		
Mean ± SD (range)	60.6 ± 12.0	(24–91)
≥ 45	704	(88.0)
< 45	96	(12.0)
Pathological tumor size (mm)		
Mean ± SD (range)	34.4 ± 22.3	(0.8–150.0)
Pathological tumor invasive size (mm)		
Mean ± SD (range)	21.0 ± 16.6	(0.1–150.0)
Pathological T factor		
pT1	489	(61.1)
pT2	273	(34.1)
pT3	38	(4.8)
Pathological N factor		
pN0	559	(69.9)
pN1	176	(22.0)
pN2	37	(4.6)
pN3	28	(3.5)
Lymphatic invasion		
Positive	375	(46.9)
Negative	425	(53.1)
Histological grade		
Grade I	271	(33.9)
Grade II	250	(31.3)
Grade III	279	(34.8)
Nuclear grade		
Grade 1	270	(33.8)
Grade 2	220	(27.5)
Grade 3	310	(38.7)
Estrogen receptor		
Positive	636	(79.5)
Negative	164	(20.5)
Progesterone receptor		
Positive	565	(70.6)
Negative	235	(29.4)
HER2		
Positive	97	(12.1)
Negative	703	(87.9)
Ki-67 labeling index (%)		
Mean ± SD (range)	16.8 ± 16.1	(0.0–90.0)
≥ 14	329	(41.1)
< 14	435	(54.4)
No data	36	(4.5)
Histological type		
Invasive carcinoma of no special type (Invasive ductal carcinoma)	672	(83.9)
Invasive lobular carcinoma	51	(6.4)
Tubular carcinoma	2	(0.3)
Mucinous carcinoma	32	(4.0)
Invasive micropapillary carcinoma	9	(1.1)
Carcinoma with apocrine differentiation (apocrine carcinoma)	21	(2.6)
Metaplastic carcinoma	7	(0.9)
Carcinoma with medullary features (medullary carcinoma)	3	(0.4)
Adenoid cystic carcinoma	1	(0.1)
Paget disease	2	(0.3)
UICC AS		
I	401	(50.1)
II	324	(40.5)
III	75	(9.4)
AJCC PS		
I	535	(66.8)
II	163	(20.4)
III	102	(12.8)
PS using nuclear grade (PS-NG)		
I	534	(66.7)
II	159	(19.9)
III	107	(13.4)
Procedure (breast)		
Mastectomy	468	(58.5)
Breast conserving surgery	332	(41.5)
Procedure (axillary lymph node)		
SNB	519	(64.8)
SNB → Ax	139	(17.4)
Ax	135	(16.9)
No therapy	7	(0.9)
Endocrine therapy		
Yes	572	(71.5)
No	185	(23.1)
No data	43	(5.4)
Chemotherapy		
Yes	277	(34.6)
No	486	(60.8)
No data	37	(4.6)
Anti-HER2 therapy		
Yes	19	(2.4)
No	744	(93.0)
No data	37	(4.6)
Radiation therapy		
Residual breast irradiation	278	(34.8)
Postmastectomy radiation therapy	35	(4.4)
Not done	455	(56.8)
No data	32	(4.0)
Relapse-free survival rate (%)		
5-year	89.5	
10-year	82.8	
Overall survival rate (%)		
5-year	96	
10-year	88.5	

*Ax* axillary lymph node dissection, *AJCC PS* American Joint Committee on Cancers prognostic stage, *ER* estrogen receptor, *HER2* human epidermal growth factor receptor 2, *SD* standard deviation, *SNB* sentinel lymph node biopsy, *UICC AS* Union for International Cancer Control anatomical staging

Pathological AJCC PS of the patients was I in 535 (66.8%), II in 163 (20.4%), and III in 102 (12.8%). Pathological UICC AS of the patients was I in 401 (50.1%), II in 324 (40.5%), and III in 75 (9.4%). The ratio of patients with AJCC PS stage I and stage III was significantly higher than that with UICC AS stage I and stage III (*P* < 0.0001).

Pathological PS-NG of the patients was I in 534 (66.7%), II in 159 (19.9%), and III in 107 (13.4%). The ratio of patients with PS-NG stage I and stage III was significantly higher than that with UICC AS stage I and stage III (*P* < 0.0001).

Of the 800 patients, the 5-year and 10-year RFS rates were 89.5 and 82.8%, respectively (median follow-up 5.5 years), and the 5-year and 10-year OS rates were 96.0 and 88.5%, respectively (median follow-up 6.0 years).

### Comparison of survival curves

**Fig. 1:**
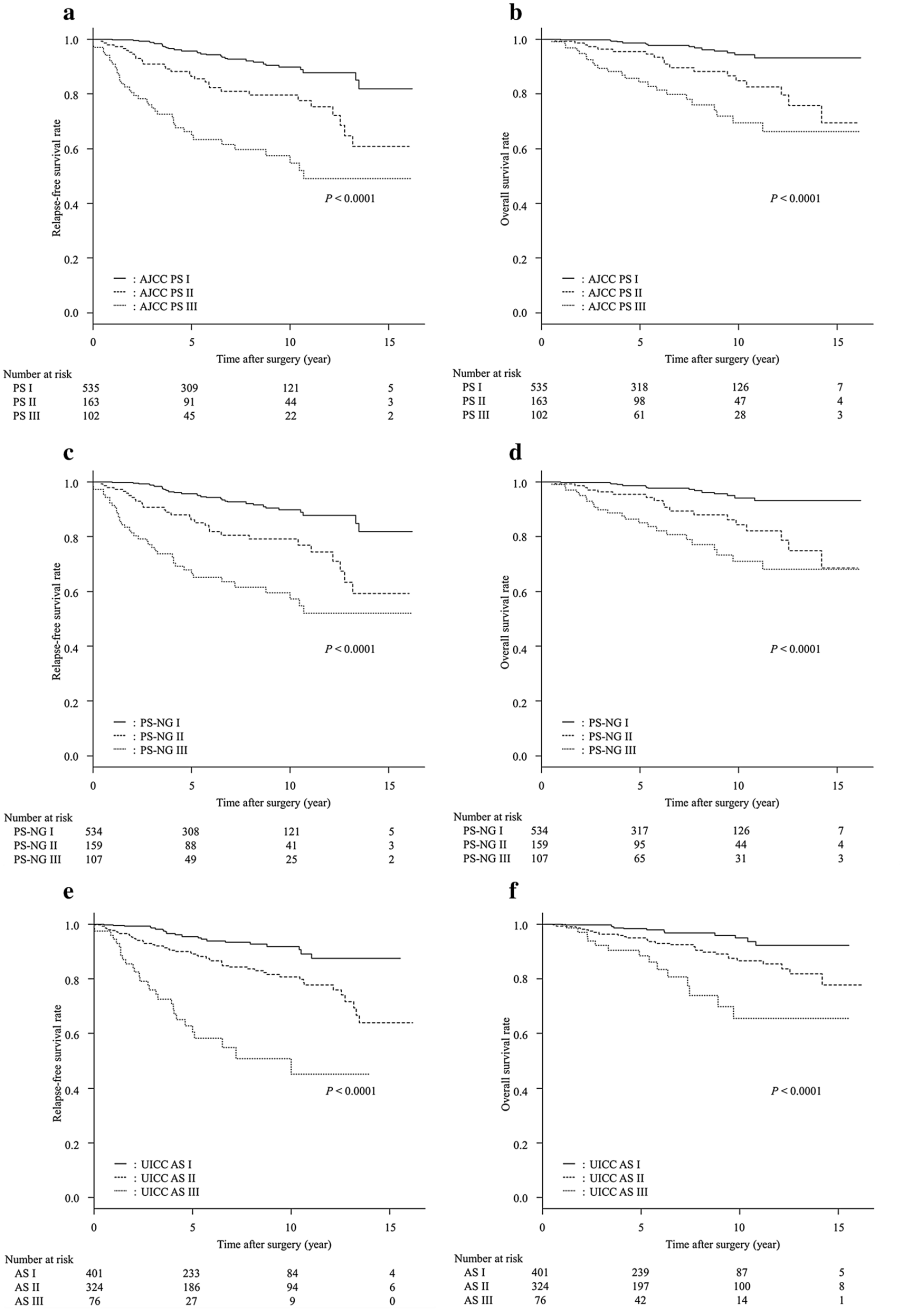
Relapse-free survival (RFS) curves and overall survival (OS) curves of 800 patients with breast cancer stratified by (**a**, **b**) American Joint Committee on Cancer (AJCC) prognostic stage (PS), (**c**, **d**) PS using nuclear grade (NG) instead of histological grade (PS-NG), and (**e**, **f**) Union for International Cancer Control (UICC) anatomical stage (AS). In **a**, **c**, **e**, three RFS curves were significantly different (*P* < 0.0001, each). In **b**, **d**, **f**, three OS curves were significantly different (*P* < 0.0001, each)

The RFS and OS curves of all 800 patients, stratified by AJCC PS, PS-NG, and UICC AS are shown in Fig. 1. Both RFS and OS curves were significantly different among the AJCC PS I, II, and III groups (*P* < 0.0001, each) (Fig. 1a, b). In AJCC PS I, II, and III groups, the 5-year RFS rates were 95.6, 86.5, and 64.8%, respectively, while the 10-year RFS rates were 89.7, 79.6, and 54.8%, respectively. Likewise, in AJCC PS I, II, and III groups, the 5-year OS rates were 98.7, 95.5, and 84.3%, respectively, while the 10-year OS rates were 94.2, 84.8, and 69.5%, respectively. With regard to AJCC PS, the AIC and the C-index for RFS were 1184.8 and 0.730, and those for OS were 629.8 and 0.736, respectively.

In the same way, both RFS and OS curves were significantly different among the PS-NG I, II, and III groups (*P* < 0.0001, each) (Fig. 1c, d). In PS-NG I, II, and III groups, the 5-year RFS rates were 95.6, 86.2, and 66.5%, respectively, while the 10-year RFS rates were 89.7, 79.1, and 57.1%, respectively. Likewise, in PS-NG I, II, and III groups, the 5-year OS rates were 98.7, 95.4, and 85.0%, respectively, while the 10-year OS rates were 94.2, 84.3, and 71.0%, respectively. With regard to PS-NG, the AIC and the C-index for RFS were 1188.4 and 0.727, and those for OS were 631.5 and 0.733, respectively.

There was a significant difference in the RFS and OS curves among the AS I, II, and III groups (*P* < 0.0001, each) (Fig. 1e, f). In UICC AS I, II, and III groups, the 5-year RFS rates were 95.4, 89.1, and 59.9%, respectively, while the 10-year RFS rates were 91.8, 80.8, and 44.7%, respectively. Likewise, in AS I, II, and III groups, the 5-year OS rates were 98.3, 95.0, and 88.2%, respectively, while the 10-year OS rates were 94.9, 86.6, and 65.4%, respectively. With regard to the UICC AS, the AIC and the C-index for RFS were 1192.9 and 0.699, while those for OS were 645.1 and 0.679, respectively. The AICs were lower and the C indices were higher in AJCC PS groups and in PS-NG groups than in the UICC AS groups.

**Fig. 2:**
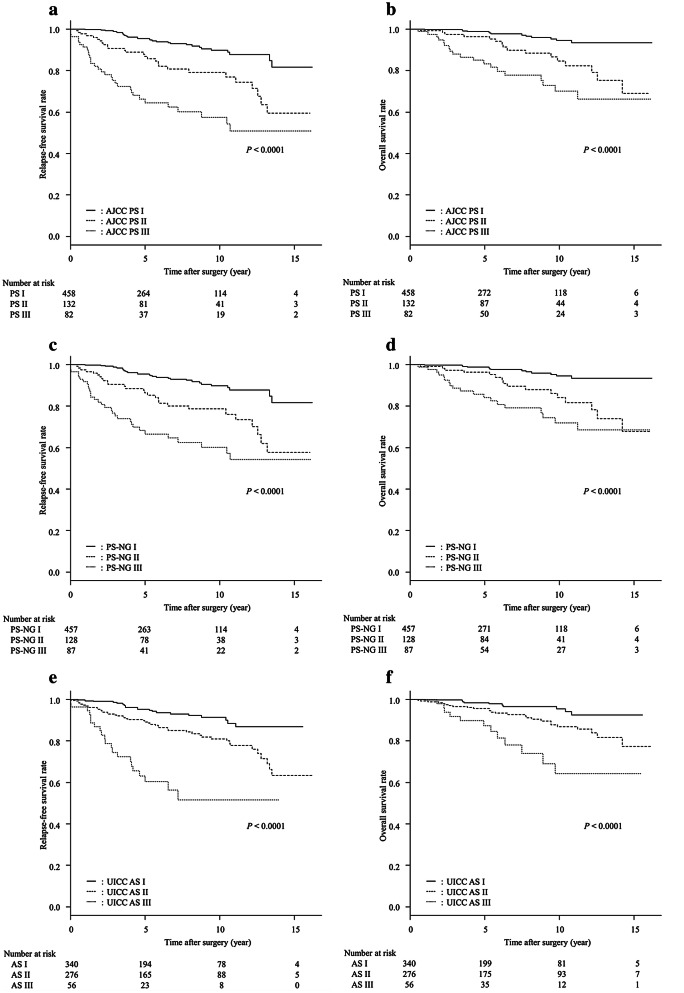
Relapse-free survival (RFS) curves and overall survival (OS) curves of 672 patients with invasive carcinoma of no special type stratified by (**a**, **b**) American Joint Committee on Cancer (AJCC) prognostic stage (PS), (**c**, **d**) PS using nuclear grade (NG) instead of histological grade (PS-NG), and (**e**, **f**) Union for International Cancer Control (UICC) anatomical stage (AS). In **a**, **c**, **e**, three RFS curves were significantly different (*P* < 0.0001, each). In **b**, **d**, **f**, three OS curves were significantly different (*P* < 0.0001, each)

The RFS and OS curves of 672 patients with invasive carcinoma of no special types (invasive ductal carcinoma), stratified with AJCC PS, PS-NG, and UICC AS, are shown in Fig. 2. In the 672 cases stratified with the AJCC PS, the AIC and the C-index for RFS were 985.9 and 0.724, while those for OS were 524.4 and 0.735, respectively. For these 672 cases stratified with the PS-NG, the AIC and the C-index for RFS were 989.3 and 0.720, while those for OS were 526.1 and 0.731, respectively. On the other hand, with regard to the stratification with UICC AS, the AIC and the C-index for RFS were 998.3 and 0.685, while those for OS were 536.6 and 0.680, respectively. The AICs were lower and the C indices were higher in AJCC PS groups or in PS-NG groups than in the UICC AS groups.

### Univariate and multivariate analyses

Cox’s univariate analyses were performed to estimate the risk of recurrence in patients with the following six clinicopathological parameters: pT, pN, HG, ER, PgR, and HER2 (Table 2). Of these, four parameters (pT, pN, HG, and PgR) were significant risk factors of recurrence (*P* < 0.0001, < 0.0001, < 0.0001, and 0.0310, respectively). ER only showed a marginal significance (*P* = 0.0729). In the Cox’s multivariate analysis including the parameters that were significant in the univariate analyses, pT, pN, and HG were independent prognostic factors of RFS (*P* = 0.0003, < 0.0001, and < 0.0001, respectively). Results from the same analyses including NG instead of HG showed almost the same results (Supplementary Table 1).

**Table 2:** Results of the Cox’s univariate and multivariate proportional hazard model analyses using clinicopathological factors for relapse-free survival (*n* = 800)

Parameter (unfavorable vs. favorable)	Univariate analysis			Multivariate analysis		
	Hazard ratio	95% CI	*P* value	Hazard ratio	95% CI	*P* value
Pathological T factor (pT2, pT3 vs. pT1)	3.52	2.35–5.40	< 0.0001	2.24	1.44–3.53	0.0003
Pathological N factor (pN3 vs. pN0, pN1, pN2)	8.98	5.04–15.0	< 0.0001	4.46	2.44–7.75	< 0.0001
Histological grade (Grade III vs. Grade I, Grade II)	3.64	2.44–5.53	< 0.0001	2.55	1.63–4.05	< 0.0001
Estrogen receptor (negative vs. positive)	1.50	0.96–2.28	0.0729			
Progesterone receptor (negative vs. positive)	1.56	1.04–2.31	0.0310	1.04	0.68–1.58	0.865
HER2 (positive vs. negative)	1.11	0.62–1.86	0.713			

*CI* confidence interval, *HER2* human epidermal growth factor receptor 2

In the same way, Cox’s univariate analyses were also performed to estimate the risk of death in patients with the aforementioned six clinicopathological parameters (Table 3). Of these, five parameters (pT, pN, HG, ER, and PgR) were significant risk factors of death (*P* < 0.0001, 0.0001, < 0.0001, 0.0156, and 0.0092, respectively). In the Cox’s multivariate analysis including the parameters that were significant in univariate analyses, pT, pN, and HG were independent prognostic factors of OS (*P* = 0.0001, 0.0193, and 0.0244, respectively). PgR was significant in the univariate analysis, but was excluded from the multivariate analysis because of its collinearity with ER. Results from the same analyses employing NG instead of HG showed almost the same results (Supplementary Table 2).

**Table 3:** Results of the Cox’s univariate and multivariate proportional hazard model analyses using clinicopathological factors for overall survival (*n* = 800)

Parameter (unfavorable vs. favorable)	Univariate analysis			Multivariate analysis		
	Hazard ratio	95% CI	*P* value	Hazard ratio	95% CI	*P* value
Pathological T factor (pT2, pT3 vs. pT1)	4.58	2.56–8.70	< 0.0001	3.22	1.74–6.30	0.0001
Pathological N factor (pN3 vs. pN0, pN1, pN2)	5.44	2.49–10.6	0.0001	2.67	1.19–5.40	0.0193
Histological grade (Grade III vs. Grade I, Grade II)	3.54	2.05–6.36	< 0.0001	2.04	1.10–3.90	0.0244
Estrogen receptor (negative vs. positive)	2.02	1.15–3.45	0.0156	1.37	0.75–2.45	0.297
Progesterone receptor (negative vs. positive)	2.04	1.20–3.45	0.0092			
HER2 (positive vs. negative)	1.21	0.55–2.36	0.606			

*CI* confidence interval, *HER2* human epidermal growth factor receptor 2

### Discordance between of UICC AS and AJCC PS

**Fig. 3:**
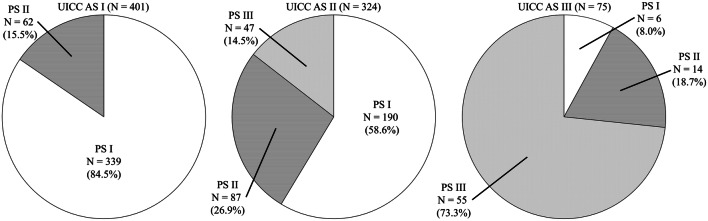
Concordance between Union for International Cancer Control (UICC) anatomical stage (AS) and the American Joint Committee on Cancer (AJCC) prognostic stage (PS). In UICC AS I patient group and AS III patient group, the stages were concordant in 84.5 and 73.3% of cases between AS and PS, respectively. By contrast, in UICC AS II group, the stage was discordant between AS and PS in 73.1% of cases

Each UICC AS group was stratified by AJCC PS (Fig. 3). In the 401 patients at UICC AS I, 339 patients (84.5%) were at AJCC PS I, 62 (15.5%) patients were at AJCC PS II, and no patient was at AJCC PS III. In the 324 patients at UICC AS II, 190 patients (58.6%) were at AJCC PS I, 87 (26.9%) patients were at AJCC PS II, and 47 patients (14.5%) were at AJCC PS III. In the 75 patients at UICC AS III, six patients (8.0%) were at AJCC PS I, 14 patients (18.7%) were at AJCC PS II, and 55 patients (73.3%) were at AJCC PS III. The concordance rate in stage between AS and PS was 60.1%. Results from the same analyses between UICC AS and PS-NG showed almost the same results (Supplementary Fig. 1). The concordance rate in stage between AS and PS-NG was 59.6%.

### Prognostic significance of AJCC PS–UICC AS discordance cases

**Fig. 4:**
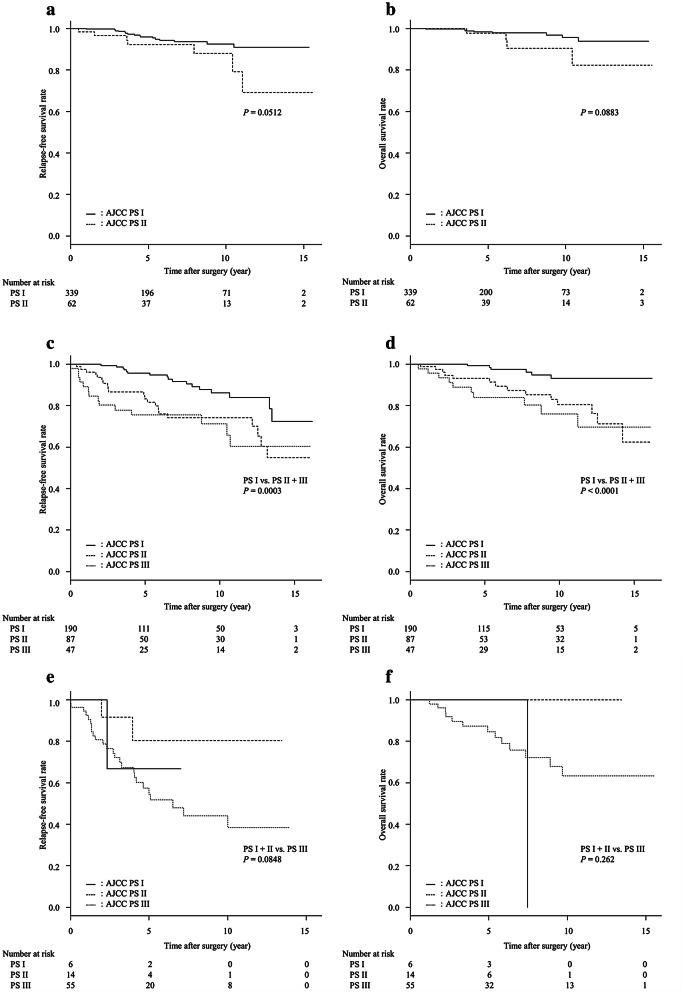
The relapse-free survival (RFS) curves (**a**) and overall survival (OS) curves (**b**) of 401 patients with UICC AS I breast cancer stratified by AJCC PS status. **a** Curves for AJCC PS I and PS II subgroups tended to differ but were not of statistical significance (*P* = 0.0512). **b** Curves tended to differ but were not of statistical significance (*P* = 0.0883). The RFS curves (**c**) and OS curves (**d**) for 324 patients with UICC AS II breast cancer stratified by AJCC PS status. (**c**) RFS curves were significantly different between AJCC PS I subgroup and PS II + III subgroup (*P* = 0.0003). **d** OS curves were significantly different between AJCC PS I subgroup and PS II + III subgroup (*P* < 0.0001). The RFS curves (**e**) and OS curves (**f**) of 75 patients with UICC AS III breast cancer stratified by AJCC PS status. **e** Curves for AJCC PS I + II subgroup and PS III subgroup tended to differ but were not significant (*P* = 0.0848). **f** Curves did not differ significantly (*P* = 0.262)

The RFS and OS curves for each UICC AS group stratified into AJCC PS groups are shown in Fig. 4. Of the 401 patients with UICC AS I, the AJCC PS II subgroup tended to show worse prognosis than the AJCC PS I subgroup in both RFS and OS (*P* = 0.0512 and *P* = 0.0883, respectively), although the differences were not significant (Fig. 3a, b). Of the 324 patients with UICC AS II, the RFS and OS curves were significantly different between AJCC PS I and AJCC PS II + III subgroup (*P* = 0.0003 for RFS and *P* < 0.0001 for OS) (Fig. 3c, d). Of the 75 patients with UICC AS III, there were no significant differences in both RFS and OS between the AJCC PS I + II subgroup and AJCC PS III subgroup (*P* = 0.0948 for RFS and *P* = 0.262 for OS) (Fig. 3e, f). For UICC AS III, the lack in statistical differences might have been due in part to the small number of cases. Results from the same analyses using PS-NG instead of AJCC PS showed almost the same results (Supplementary Fig. 2).

## Discussion

In the present study, we evaluated the usefulness of pathological AJCC PS for prediction of prognosis of primary breast cancer patients by comparing it with pathological UICC AS. First, we compared the number of cases at each stage (I, II, and III) between PS and AS. Second, we compared the survival curves of the patients at each stage (I, II, and III) between PS and AS, and tested the validity of PS compared with AS as a model and the model’s predictive performance.

We were able to confirm that the biological factors including grade, ER, PgR, and HER2 were useful not only for the selection of appropriate adjuvant systemic therapies, but also for the prediction of patient’s prognosis. The PS, which includes the AS and biological factors, appeared to be a useful model for stratifying the primary breast cancer patients into biologically distinct groups. The present results were similar to those reported by Abdel-Rahman et al. [24] and Li et al. [25].

The majority (84.5%) of AS I cases were concordant with PS I cases. Likewise, the majority (73.3%) of AS III were concordant with PS III cases. By contrast, the situation was different for stage II: of the 324 patients with AS II, 87 had PS II, and the concordance rate between AS II and PS II was only 26.9%. In other words, the number of PS I cases was 1.33 folds (535 vs. 401) as high as that of AS I, and the number of cases of PS III was 1.36 folds (102 vs. 75) as high as that of AS III. Nonetheless, between PS and AS, the survival curves of patients with stages I, II, and III were almost similar (Fig. 1).

The number of AS II cases was 1.99 folds (324 vs. 163) as high as that of PS II. In patients with AS II, the subgroups stratified by PS, i.e., PS I PS II, and PS III subgroups, showed significantly different RFS and OS curves. Therefore, many patients who should be classified as having stage I and stage III appeared to have been included in the AS II group. Furthermore, in patients with AS I, the subgroups stratified by PS, i.e., PS I and PS II subgroups, showed nearly significant difference in RFS and OS curves. Therefore, a small proportion of patients who could be classified as having stage II was included in the AS I group.

To trichotomize the patients according to RFS or OS, the PS classification system appeared to be a more accurate tool in stratifying patients than the AS classification system. The results shown in Fig. 4 exemplified this speculation. For example, in the AS II group, the prognosis of PS I subgroup was obviously better than that of the PS II + III subgroup and was similar to that of the UICC AS I patients. Likewise, even in the AS I group, the prognosis of PS II subgroup tended to be poorer than that of the PS I subgroup, although the difference was not significant (Fig. 3a, b). Furthermore, in the AS III group, the curves for PS III subgroup and PS II subgroup appeared to be different, although the difference was not significant (Fig. 3e, f). These findings were confirmed statistically using the AIC and C-index.

Because NG as well as HG has been used in Japan, we also evaluated PS using NG instead of HG. Prognostic values, differences in survival curves among stages, AIC and C-index of PS-NG were very similar with those of AJCC PS. Therefore, it appeared that PS classification employing NG was not inferior to AJCC PS for patient prognostication. NG is registered in the National Clinical Database of breast cancer in Japan but HG is not. In large scale studies, PS using NG might be able to a surrogate for AJCC PS for the comparison between PS and AS.

This study has some limitations. It is a retrospective study that was conducted in a single facility using a relatively small number of samples. We had to exclude many cases from the study because of various reasons described in the methods. Nonetheless, we were able to show that PS was a valid model for prognostication of breast cancer patients into biologically distinct groups. However, further multicenter and prospective studies are needed to confirm the effectiveness of AJCC PS.

## Conclusion

We studied the prognostic significance of AJCC PS classification in patients with primary breast cancer. AJCC PS showed lower AIC and higher C-index than UICC AS in both RFS and OS. For the prognostication of surgically resectable primary breast cancers, AJCC PS appeared to be used in the stratification of these cases more appropriately than UICC AS.

## Electronic supplementary material

Below is the link to the electronic supplementary material.

Supplementary Fig. 1 Concordance between Union for International Cancer Control (UICC) anatomical stage (AS) and the prognostic stage using nuclear grade (PS-NG). In UICC AS I patient group and AS III patient group, the stages were concordant in 84.3% and 74.7% of cases between AS and PS-NG, respectively. By contrast, in UICC AS II group, the stage was discordant between AS and PS-NG in 74.4% of cases (TIF 503 kb)

Supplementary Fig. 2 The relapse-free survival (RFS) curves (a) and overall survival (OS) curves (b) of 401 patients with Union for International Cancer Control (UICC) anatomical stage (AS) I breast cancer stratified by prognostic stage using nuclear grade (PS-NG). (a and b) Curves for PS-NG I and II subgroups tended to differ but were not of statistical significance [P = 0.0596 in (a); P = 0.0976 in (b)]. The RFS curves (c) and OS curves (d) for 324 patients with UICC AS II breast cancer stratified by PS-NG. RFS curves and OS curves were significantly different between PS-NG I subgroup and PS-NG II + III subgroup [P = 0.0003 in (c); P < 0.0001 in (d)]. The RFS curves (e) and OS curves (f) of 75 patients with UICC AS III breast cancer stratified by PS-NG. (e) Curves for PS-NG I + II subgroup and PS-NG III subgroup tended to differ but were not significant (P = 0.127). (f) Curves did not differ significantly (P = 0.299) (TIF 583 kb)

Supplementary file3 (DOCX 17 kb)
