# Supplementary figures and images for: Comparison between AJCC 8th prognostic stage and UICC anatomical stage in patients with primary breast cancer: a single institutional retrospective study
Source: Breast Cancer. 2020 Jun 3;27(6):1114–25. doi: 10.1007/s12282-020-01115-x (PMC7567685; doi:10.1007/s12282-020-01115-x)

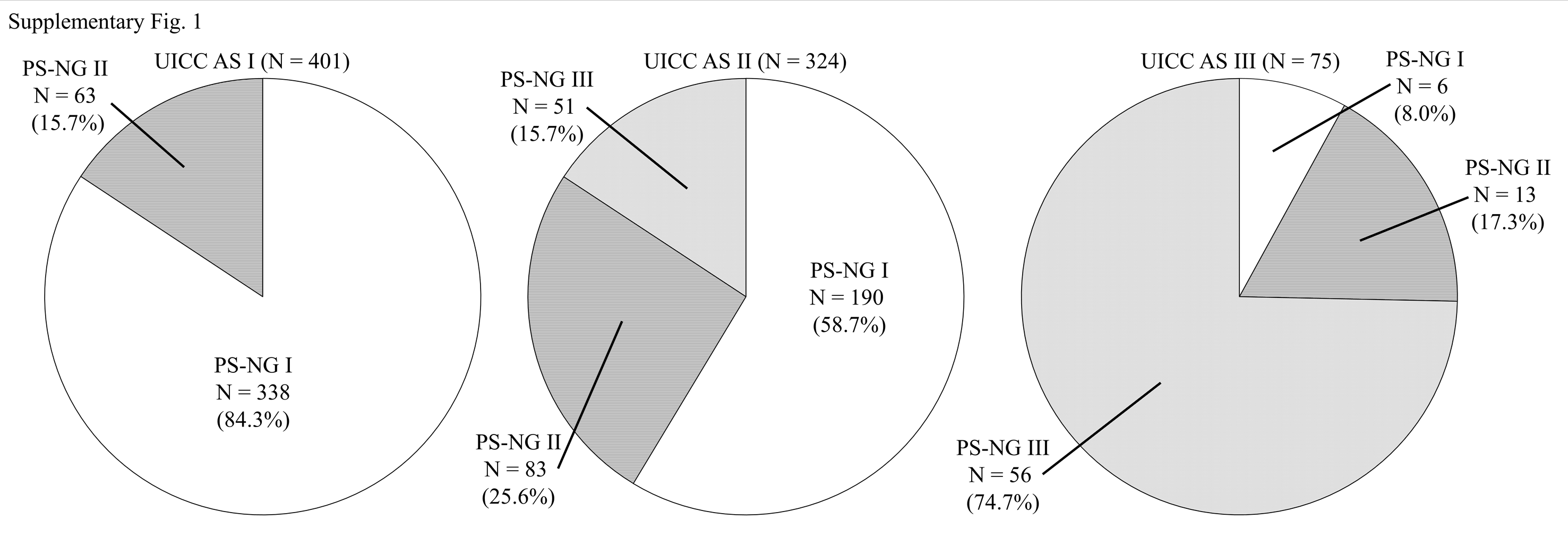

Supplement: Supplementary file 1 — Supplementary Fig. 1 Concordance between Union for International Cancer Control (UICC) anatomical stage (AS) and the prognostic stage using nuclear grade (PS-NG). In UICC AS I patient group and AS III patient group, the stages were concordant in 84.3% and 74.7% of cases between AS and PS-NG, respectively. By contrast, in UICC AS II group, the stage was discordant between AS and PS-NG in 74.4% of cases (TIF 503 kb) [file 12282_2020_1115_MOESM1_ESM.tif]

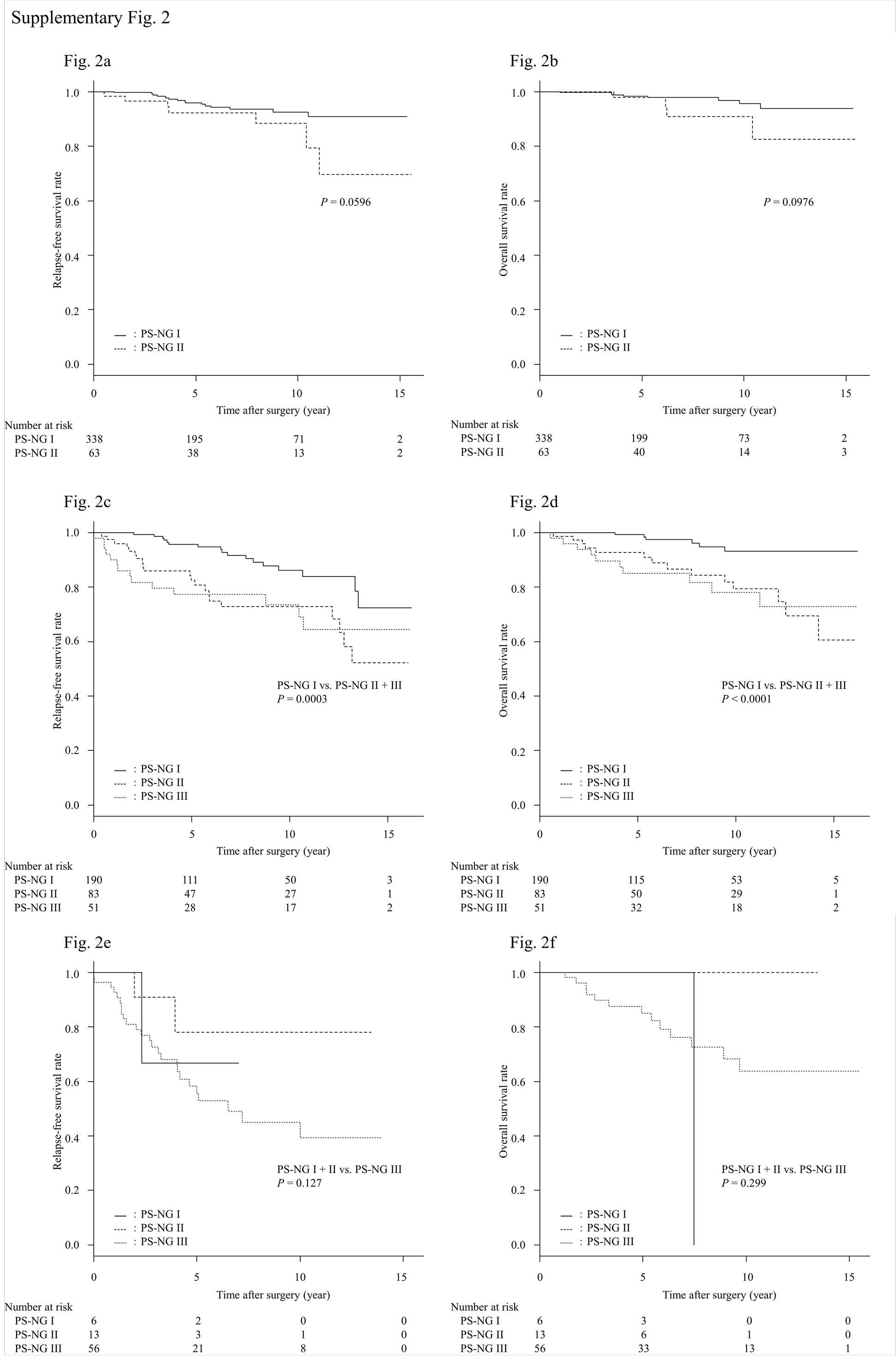

Supplement: Supplementary file 2 — Supplementary Fig. 2 The relapse-free survival (RFS) curves (a) and overall survival (OS) curves (b) of 401 patients with Union for International Cancer Control (UICC) anatomical stage (AS) I breast cancer stratified by prognostic stage using nuclear grade (PS-NG). (a and b) Curves for PS-NG I and II subgroups tended to differ but were not of statistical significance [P = 0.0596 in (a); P = 0.0976 in (b)]. The RFS curves (c) and OS curves (d) for 324 patients with UICC AS II breast cancer stratified by PS-NG. RFS curves and OS curves were significantly different between PS-NG I subgroup and PS-NG II + III subgroup [P = 0.0003 in (c); P < 0.0001 in (d)]. The RFS curves (e) and OS curves (f) of 75 patients with UICC AS III breast cancer stratified by PS-NG. (e) Curves for PS-NG I + II subgroup and PS-NG III subgroup tended to differ but were not significant (P = 0.127). (f) Curves did not differ significantly (P = 0.299) (TIF 583 kb) [file 12282_2020_1115_MOESM2_ESM.tif]
