# Supplementary material for: Comparison between AJCC 8th prognostic stage and UICC anatomical stage in patients with primary breast cancer: a single institutional retrospective study
Source: Breast Cancer. 2020 Jun 3;27(6):1114–25. doi: 10.1007/s12282-020-01115-x (PMC7567685; doi:10.1007/s12282-020-01115-x)
Supplement: Supplementary file 3 — Supplementary file3 (DOCX 17 kb) [file 12282_2020_1115_MOESM3_ESM.docx]

| Supplementary Table 1. Cox’s univariate and multivariate proportional hazard model analyses using clinicopathological factors including nuclear grade for relapse-free survival (n = 800) | | | | | | | |
| --- | --- | --- | --- | --- | --- | --- | --- |
| Parameter | Univariate analysis | | |  | Multivariate analysis | | |
| (Unfavorable vs. favorable) | Hazard ratio | 95% CI | *P* value |  | Hazard ratio | 95% CI | *P* value |
| Pathological T factor | 3.52 | 2.35–5.40 | < 0.0001 |  | 2.33 | 1.50–3.68 | 0.0001 |
| (pT2, pT3 vs. pT1, pTis) |  |  |  |  |  |  |  |
| Pathological N factor | 8.98 | 5.04–15.0 | < 0.0001 |  | 4.52 | 2.46–7.85 | < 0.0001 |
| (pN3 vs. pN0, pN1, pN2) |  |  |  |  |  |  |  |
| Nuclear grade | 3.27 | 2.18–5.01 | < 0.0001 |  | 2.23 | 1.41–3.59 | 0.0006 |
| (Grade 3 vs. Grade 1 and Grade 2) |  |  |  |  |  |  |  |
| Estrogen receptor | 1.50 | 0.96–2.28 | 0.0729 |  |  |  |  |
| (Negative vs. positive) |  |  |  |  |  |  |  |
| Progesterone receptor | 1.62 | 1.09–2.40 | 0.0183 |  | 1.09 | 0.71–1.66 | 0.705 |
| (Negative vs. positive) |  |  |  |  |  |  |  |
| HER2 | 1.11 | 0.54–1.62 | 0.713 |  |  |  |  |
| (Positive vs. negative) |  |  |  |  |  |  |  |

CI, Confidence interval;

HER2, Human epidermal growth factor receptor 2

| Supplementary Table 2. Cox’s univariate and multivariate proportional hazard model analyses using clinicopathological factors including nuclear grade for overall survival (n = 800) | | | | | | | |
| --- | --- | --- | --- | --- | --- | --- | --- |
| Parameter | Univariate analysis | | |  | Multivariate analysis | | |
| (Unfavorable vs. favorable) | Hazard ratio | 95% CI | *P* value |  | Hazard ratio | 95% CI | *P* value |
| Pathological T factor | 4.58 | 2.56–8.70 | < 0.0001 |  | 3.27 | 1.77–6.38 | 0.0001 |
| (pT2, pT3 vs. pT1, pTis) |  |  |  |  |  |  |  |
| Pathological N factor | 5.44 | 2.49–10.5 | 0.0001 |  | 2.72 | 1.21–5.50 | 0.0175 |
| (pN3 vs. pN0, pN1, pN2) |  |  |  |  |  |  |  |
| Nuclear grade | 3.48 | 1.98–6.39 | < 0.0001 |  | 2.01 | 1.05–3.97 | 0.0360 |
| (Grade 3 vs. Grade 1 and Grade 2) |  |  |  |  |  |  |  |
| Estrogen receptor | 2.02 | 1.15–3.45 | 0.0156 |  | 1.33 | 0.72–2.42 | 0.350 |
| (Negative vs. positive) |  |  |  |  |  |  |  |
| Progesterone receptor | 2.09 | 1.23–3.54 | 0.0066 |  |  |  |  |
| (Negative vs. positive) |  |  |  |  |  |  |  |
| HER2 | 1.21 | 0.55–2.36 | 0.606 |  |  |  |  |
| (Positive vs. negative) |  |  |  |  |  |  |  |

CI, Confidence interval;

HER2, Human epidermal growth factor receptor 2
